# Gene mapping and molecular analysis of hereditarynon-polyposis colorectal cancer (Lynch Syndrome)using systems biological approaches

**DOI:** 10.6026/97320630015269

**Published:** 2019-04-15

**Authors:** Mahmood Rasool, Sajjad Karim, Muhammad Imran Naseer, Peter Natesan Pushparaj, Adel Abuzenadah, Mohammed Hussein Al-Qahtani

**Affiliations:** 1 Center of Excellence in Genomic Medicine Research, King Abdulaziz University, Jeddah, Saudi Arabia; 2 Department of MedicalLaboratory Technology, Faculty of Applied Medical Sciences, King Abdulaziz University, Jeddah, Saudi Arabia

**Keywords:** Lynch syndrome, HNPCC, Mismatch repair genes, Open target platform

## Abstract

Hereditary non-polyposis colorectal cancer (HNPCC) also known as Lynch Syndrome (LS), is a hereditary form of colorectal cancer (CRC). LSis caused by mutations in the mismatch repair (MMR) genes, mostly in MLH1, MSH2, MSH6 and PMS2. Identification of these gene mutations is essential to diagnose CRC, especially at a young age to increase the survival rate. Using open target platform, we have performed genetic association studies to analyze the different genes involved in the LS and to obtain target for disease evidence. We have also analyzed upstream regulators as target molecules in the data sets. We discovered that MLH1, MSH2, MSH6, PMS2, MLH3, EPCAM, TGFBR2, FBXO11 and PRSS58 were showing most association in LS. Our findings may further enhance the understanding of the hereditaryform of CRC.

## Background

Lynch syndrome is an autosomal dominant condition caused by many mismatch repair genes including four important genes; MLH1, PMS2, MSH2 and MSH6 1. LS was named in honor of Henry T. Lynch, who reported several families in detail during 1966-67 2, 3. LS accounts for 1-5% of all CRC and also present an increased risk of many extra colonic cancer types 4. Mutations in the MMR genes lead to the inactivation or lower efficiency to repair mismatches in DNA that leads to the accumulation of spontaneous mutations mostly consist of the insertions and deletions in short repetitive DNA sequences termed microsatellites. The changes in short microsatellite sequences lead to the microsatellite instability, that is found in the majority of LS tumors (>90%) in patients with germ line mutations in MMR genes 5. Therefore the current strategy before sequencing these MMR genes is to do the microsatellite instability (MSI) testing. So, if the patient tumor DNA is found with MSI, it will likely yield a mutation in MMR genes. Generally, the five different regions with microsatellites are looked at, and the tumor is considered highly unstable if instability is found in two or more regions. While the tumor is called as unstable-low if the instability is found in only one region and stable if no instability is found 6-9. The identification of the MMR gene status is very important for surveillance and early intervention especially in the carriers and the family members of the CRC patients, therefore appropriate measure could be taken to limit the disease and improve the survival of the patients and carriers of the disease. And also excluding the family members for any mismatch gene mutation carriers may reduce the worry and high-risk surveillance burden of prevention testing. In this article, we have utilized open target platform for genetic association studies of different genes spectrum involved in LS, their upstream regulators and canonical pathways

## Methodology

### Data-Mining of Genetic Associations in Lynch Syndrome:

We have used the Open Targets Platform (https://www.targetvalidation.org/),a free-online integrated web resource of genetics, omics and chemical data to aid systematic drug target identification and ranking linking these associations back to the underlying evidence and its source which gives the prioritization of drugs for gene targets based on the strength of their association with a disease such as LS 10, 11. The open targets platform assemble data types from multiple open sources and implement a scoring system on the gene target-disease associations aiming at providing users to classify, recognize, and prioritize suitable drug targets for further examination. The Open Targets score for the associations is a range between 0 (no association) and 1(strong association). The Open Targets Platform gives scores with varying shades of blue (the darker the blue, the stronger the genetic association with a particular disease) and the overall association score is the result of the combination of all data source scores 10,11.

### Ingenuity Pathway Analysis:

The Knowledgebase in Ingenuity Pathway Analysis (IPA) software (Qiagen, USA) was used to obtain the list of genes implicated in the LS. The canonical pathways, upstream regulators, and the differential regulation of gene networks in the LS were further deduced by applying the Fisher's Exact Test (P<0.05) in IPA. The - log P values were plotted in the x-axis and the differentially expressed canonical pathways in the y-axis to derive top canonical pathways implicated in the LS.

## Results

**Figure 1:**
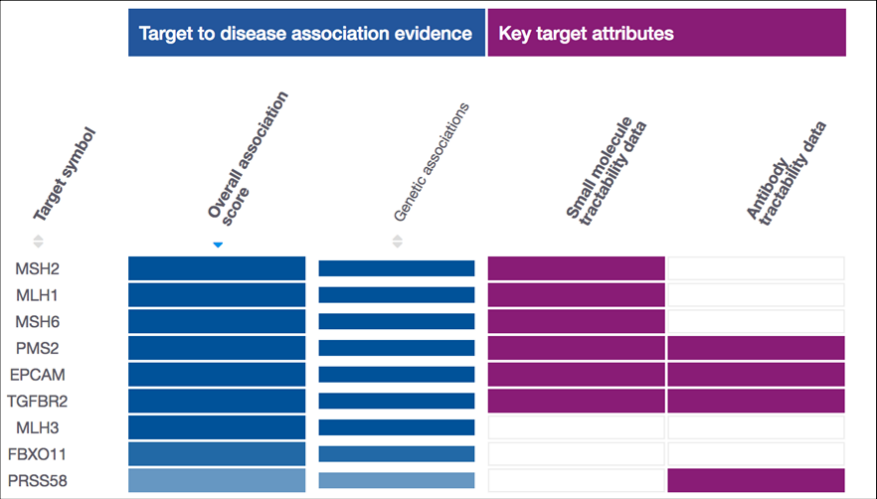
Top gene targets linked with the Lynch Syndrome was obtained from the Open Targets Platform based on the genetic association score (Darker the blue more the genetic association with Lynch Syndrome).

The results of the study are summarized in Table 1 and Table 2 and Figure 1 to Figure 3. The most genes associated with LS were found to be the MLH1, MSH2, MSH6, PMS2, EPCAM, TGFBR2, MLH3, FBXO11, and PRSS58. The most of the mutations were reported in MLH1 gene (n= 731, 589pathogenic,and 142likely pathogenic), followed by MSH2 (n=653, 546 pathogenic and 107 likely pathogenic); MSH6 (n=414, 367 pathogenic and 47 likely pathogenic); PMS2 (n=185, 144 pathogenic and 41 likely pathogenic); EPCAM (n=10, 8 pathogenic and 2 likely pathogenic); PRSS58 (n=7, 4 pathogenic and 3 likely pathogenic); MLH3 (n=3, 2 pathogenic and 1 likely pathogenic) and TGFBR2 (one pathogenic only).

## Discussion

Lynch syndrome is the most common hereditary CRC that account for more than 3% of all the colon cancer cases 12. The genetic heterogeneity of this syndrome is related to the mutations in different genes especially in four mismatch repair genes; MLH1, MSH2, MSH6, and PMS2. The mismatch repair genes contributeto various cellular functions including repairing double-stranded DNA breaks, repairing or errors during DNA synthesis, antirecombination and destabilization of DNA and apoptosis. MMR proteins serve the job of maintenance of genetic material thereforevital for the regulation of the cellular cycle. When the MMR protein is defective or lost altogether, it decreases apoptosis and increases cell survival. This leads to the selective growth advantage to the cells that lead to the more susceptibility to tissue-specific cancers 12. 

**Figure 2:**
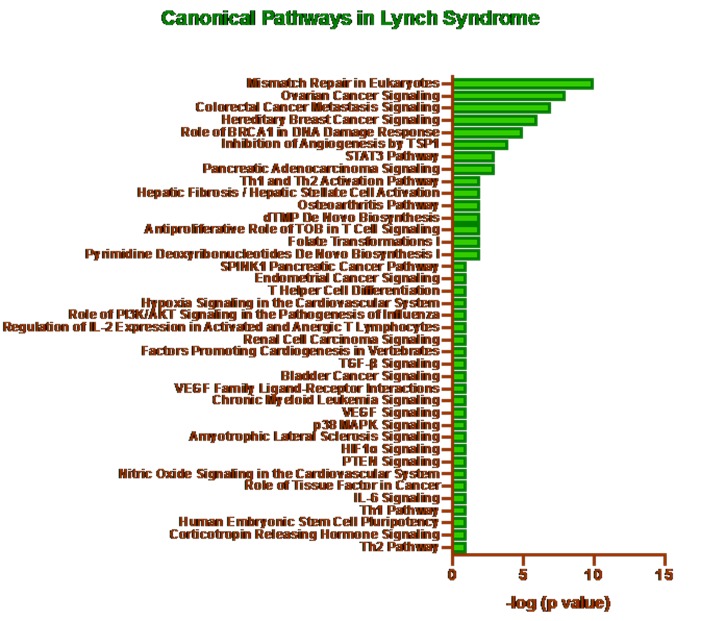
Canonical Pathways in Lynch Syndrome.The canonical pathways, in the Lynch syndrome, were identified using the IPA. The - log P values were plotted in the x-axis and the differentially expressed canonical pathways in the y-axis to derive top canonical pathways such as mismatch repair signaling implicated in the Lynch Syndrome.

**Figure 3:**
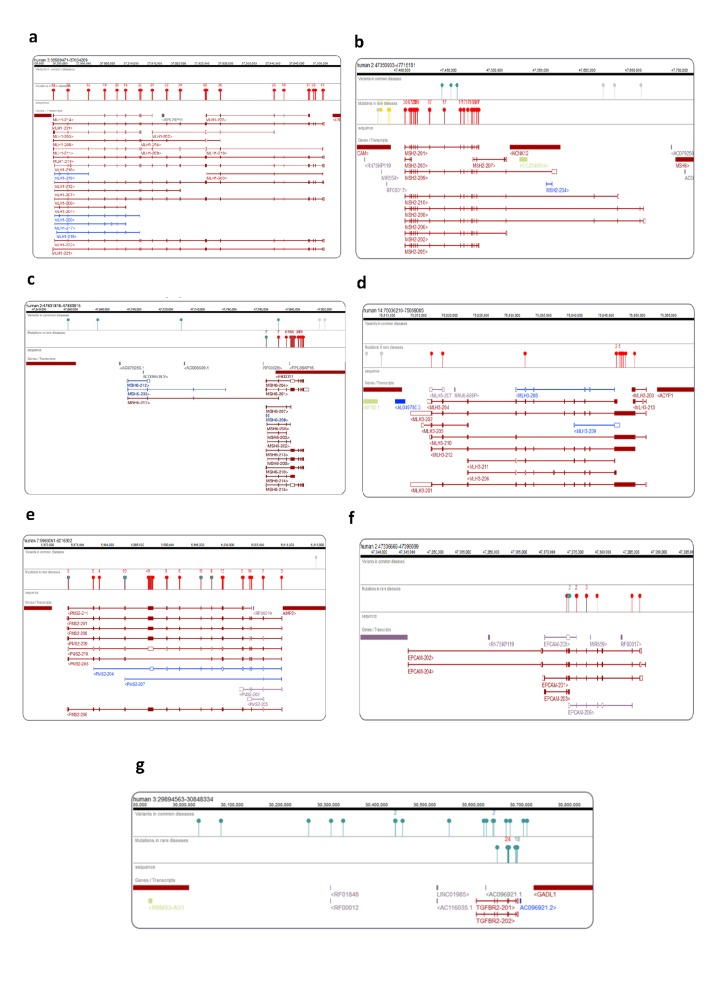
The pathogenic genetic variants in observed in MLH1, MSH2, MSH6, PMS2, MLH3, EPCAM, and TGFBR2 genes associated with Lynch syndrome are shown in the genome browser.

The mutations in MLH1 are mostly present in the LS cases (around 50% of families), while the rest MMR genes mutations account for 40-50% of the syndrome. Different approaches have been utilized so far to detect mutations underlying this syndrome specifically in these four MMR genes (MLH1, MSH2, MSH6 and PMS2), which includes Next-GenerationSequencing based targeted sequencing, Multiplex Ligation-dependent Probe Amplification, Sanger Sequencing and array-CGH 13-15. In our research, we have utilized Open Targets Platform (https://www.targetvalidation.org/) that allowed the prioritization of the genes based on the strength of their association with LS. The most important genes for the hereditary LS cancer were found to be the MLH1, MSH2, and MSH6. Together they account for more than 90% of the mutations in LS. MLH1 was on top with 731 mutations (589 pathogenic and 142 likely pathogenic), followed by MSH2 with 653 mutations (546 pathogenic and 107 likely pathogenic) and MSH6 with 414 mutations (367 pathogenic and 47 likely pathogenic). While the rest other genes including PMS2, MLH3, EPCAM, PRSS58 and TGFBR2 accounted for less than 10% of total mutations underlying LS. Canonical pathways in LSalso confirmed MMR involvement in syndrome, while other pathways included colorectal cancer metastasis signaling and ovarian cancer signaling (Figure 2).While the analysis of upstream regulators of the target molecules yield to be the MBD4, PTTG1, CHI3L1, TP53,and MYC (Table 2 shows complete list).

## Conclusion

The current study has highlighted the mutation spectrum of different genes involved in Lynch syndrome and their association with different upstream regulators and involvement in canonical pathways. This study will further pave the way to accumulate all the data and genetic studies together for better prognostic and treatment options.

## Conflict of Interest

Authors declare no conflict of interest.

## Figures and Tables

**Table 1:** Genetic associations observed in Lynch Syndrome

Gene Symbol	Gene Target ID	Overall Score	Genetic Association Score	Somatic Mutation Score	Gene Name
MLH1	ENSG00000076242	1	1	0	mutL homolog 1
MSH2	ENSG00000095002	1	1	0	mutS homolog 2
MSH6	ENSG00000116062	1	1	0	mutS homolog 6
PMS2	ENSG00000122512	1	1	0	PMS1 homolog 2, mismatch repair system component
EPCAM	ENSG00000119888	1	1	0	epithelial cell adhesion molecule
TGFBR2	ENSG00000163513	1	1	0.694444445	transforming growth factor beta receptor 2
MLH3	ENSG00000119684	1	1	0	mutL homolog 3
FBXO11	ENSG00000138081	0.834652	0.83125	0	F-box protein 11
PRSS58	ENSG00000258223	0.498263889	0.498263889	0	serine protease 58

**Table 2:** Upstream regulators and the corresponding target genes observed in Lynch syndrome

S. No	Upstream Regulator	Molecule Type	p-value of overlap	Target Genes
1	MBD4	enzyme	3.23E-13	MLH1,MSH2,MSH6,PMS2
2	PTTG1	transcription regulator	2.11E-12	MLH1,MLH3,PMS1,PMS2,Vegf,VEGFA
3	CHI3L1	enzyme	0.000000379	TGFBR2,Vegf,VEGFA
4	TP53	transcription regulator	0.000000596	MLH1,MSH2,MSH6,PMS2,TGFBR2,TYMS,Vegf,VEGFA
5	MYC	transcription regulator	0.000000924	CD274,EPCAM,MSH2,TGFBR2,TYMS,Vegf,VEGFA
6	FBLN2	other	0.00000182	Vegf, VEGFA
7	CCNA1	other	0.00000182	Vegf, VEGFA
8	IL18	cytokine	0.00000231	MSH2, TGFBR2,Vegf,VEGFA
9	SRSF6	other	0.00000303	Vegf, VEGFA
10	PDGFD	growth factor	0.00000454	Vegf, VEGFA
11	SP3	transcription regulator	0.00000533	MSH6,TGFBR2,Vegf,VEGFA
12	E2F1	transcription regulator	0.00000539	MLH1,MSH2,TYMS,Vegf,VEGFA
13	EGLN1	enzyme	0.00000599	TGFBR2,Vegf,VEGFA
14	KRAS	enzyme	0.00000666	CD274,EPCAM,TGFBR2,Vegf,VEGFA
15	F3	Trans membrane receptor	0.0000072	TGFBR2,Vegf,VEGFA
16	Cpla2	group	0.00000846	Vegf, VEGFA
17	IL17A	cytokine	0.0000085	CD274,TGFBR2,Vegf,VEGFA
18	SP1	transcription regulator	0.0000101	MSH6,TGFBR2,TYMS,Vegf,VEGFA
19	MLH1	enzyme	0.0000109	MLH1,PMS2
20	IL18BP	other	0.0000109	Vegf, VEGFA
21	LDL	complex	0.0000129	MLH1,TGFBR2,Vegf,VEGFA
22	CYP4F2	enzyme	0.0000136	Vegf, VEGFA
23	HOXB7	transcription regulator	0.0000136	Vegf, VEGFA
24	S100A7	other	0.0000166	Vegf, VEGFA
25	NFkB (complex)	complex	0.0000184	CD274,EPCAM,MSH2,Vegf,VEGFA
26	miR-150-5p (and other miRNAs w/seed CUCCCAA)	mature micro RNA	0.0000199	Vegf, VEGFA
27	SERPINB5	other	0.0000235	Vegf, VEGFA
28	HHEX	transcription regulator	0.0000275	Vegf, VEGFA
29	mir-155	Micro RNA	0.000029	MLH1,MSH2,MSH6
30	MET	kinase	0.0000314	CD274,Vegf,VEGFA
31	NREP	other	0.0000317	TGFBR2,VEGFA
32	RLN2	other	0.0000317	Vegf, VEGFA
33	PI3K (family)	group	0.0000323	CD274,Vegf,VEGFA
34	CBX4	transcription regulator	0.0000362	MSH2,VEGFA
35	APEX1	enzyme	0.000041	Vegf, VEGFA
36	ALOX12	enzyme	0.0000461	TGFBR2,VEGFA
37	PAEP	other	0.0000461	CD274,VEGFA
38	Angio tensin II receptor type 1	group	0.0000515	Vegf,VEGFA
39	HMOX1	enzyme	0.0000515	CD274,Vegf,VEGFA
40	TP73	transcription regulator	0.0000516	MLH1,MUTYH,Vegf,VEGFA
41	FOXM1	transcription regulator	0.0000576	TGFBR2,Vegf,VEGFA
42	RAS	group	0.0000602	CD274,Vegf,VEGFA
43	VHL	transcription regulator	0.0000602	TYMS, Vegf, VEGFA
44	SMAD7	transcription regulator	0.0000615	TGFBR2,Vegf,VEGFA
45	Histone h4	group	0.0000741	MSH2,TGFBR2,TYMS
46	HTATIP2	transcription regulator	0.0000761	Vegf, VEGFA
47	RELA	transcription regulator	0.0000808	CD274,EPCAM,Vegf,VEGFA
48	IL15	cytokine	0.0000837	CD274,TGFBR2,Vegf,VEGFA
49	IL4	cytokine	0.0000952	CD274,MSH2,TGFBR2,Vegf,VEGFA
50	miR-155-5p (miRNAs w/seed UAAUGCU)	mature micro RNA	0.0000969	MLH1,MSH2,MSH6
51	JUN	transcription regulator	0.000104	CD274,MSH2,Vegf,VEGFA
52	CTSB	peptidase	0.000105	Vegf, VEGFA
53	SP4	transcription regulator	0.000114	Vegf, VEGFA
54	PTEN	phosphatase	0.000123	CD274,TGFBR2,Vegf,VEGFA
55	BRCA1	transcription regulator	0.000128	MLH1,Vegf,VEGFA
56	IGF2	growth factor	0.000135	PMS1,Vegf,VEGFA
57	NOX4	enzyme	0.00014	Vegf, VEGFA
58	MAP2K1	kinase	0.000148	CD274,Vegf,VEGFA
59	GNAI2	enzyme	0.000149	MLH1,PMS2
60	STK11	kinase	0.000151	CD274,Vegf,VEGFA
61	HGF	growth factor	0.000156	EPCAM,TGFBR2,Vegf,VEGFA
62	THBS1	other	0.000158	Vegf, VEGFA
63	DRD2	GPCR	0.000168	TGFBR2,VEGFA
64	PAK1	kinase	0.000168	Vegf, VEGFA
65	SPP1	cytokine	0.000178	TGFBR2,Vegf,VEGFA
66	TEAD4	transcription regulator	0.000178	Vegf, VEGFA
67	miR-16-5p (and other miRNAs w/seed AGCAGCA)	mature micro RNA	0.000192	MSH2,PMS1,VEGFA
68	TGFBR2	kinase	0.000197	TGFBR2,Vegf,VEGFA
69	SFRP1	Trans membrane receptor	0.000199	EPCAM, Vegf
70	MERTK	kinase	0.000233	CD274,Vegf
71	ETS1	transcription regulator	0.000236	TGFBR2,TYMS,VEGFA
72	Jnk	group	0.000249	MSH2,TGFBR2,Vegf
73	STUB1	enzyme	0.000257	Vegf, VEGFA
74	TGFB1	growth factor	0.000265	EPCAM,MSH6,TGFBR2,TYMS,Vegf,VEGFA
75	ANGPT1	growth factor	0.00027	Vegf,VEGFA
76	Hsp27	group	0.000283	CD274,Vegf
77	HBEGF	growth factor	0.000283	Vegf, VEGFA
78	TNF	cytokine	0.000296	CD274,EPCAM,MSH2,TGFBR2,Vegf,VEGFA
79	C3AR1	GPCR	0.000323	TGFBR2,Vegf
80	BSG	transporter	0.000323	Vegf, VEGFA
81	ITGB2	Trans membrane receptor	0.000351	TGFBR2,VEGFA
82	MAPK7	kinase	0.000351	Vegf,VEGFA
83	NOTCH1	transcription regulator	0.000373	TGFBR2, Vegf,VEGFA
84	KLF4	transcription regulator	0.000377	EPCAM, Vegf, VEGFA
85	EPHB4	kinase	0.00038	TGFBR2,Vegf
86	OLR1	Trans membrane receptor	0.00038	TGFBR2,Vegf
87	mir-21	Micro RNA	0.000395	CD274,MSH2,Vegf
88	ALB	transporter	0.000395	Vegf, VEGFA
89	HDAC1	transcription regulator	0.000408	TGFBR2,TYMS,Vegf
90	DCN	other	0.000426	Vegf, VEGFA
